# Forward Prediction in the Posterior Parietal Cortex and Dynamic Brain-Machine Interface

**DOI:** 10.3389/fnint.2016.00035

**Published:** 2016-10-26

**Authors:** He Cui

**Affiliations:** ^1^Institute of Neuroscience, Chinese Academy of Sciences (CAS)Shanghai, China; ^2^Key Laboratory of Primate Neurobiology, Chinese Academy of Sciences (CAS)Shanghai, China

**Keywords:** neuroprosthetics, decoding, neuroengineering, internal model, motor control, paralysis

## Abstract

While remarkable progress has been made in brain-machine interfaces (BMIs) over the past two decades, it is still difficult to utilize neural signals to drive artificial actuators to produce predictive movements in response to dynamic stimuli. In contrast to naturalistic limb movements largely based on forward planning, brain-controlled neuroprosthetics mainly rely on feedback without prior trajectory formation. As an important sensorimotor interface integrating multisensory inputs and efference copy, the posterior parietal cortex (PPC) might play a proactive role in predictive motor control. Here it is proposed that predictive neural activity in PPC could be decoded to provide prosthetic control signals for guiding BMI systems in dynamic environments.

## Introduction

To interact with a changing world, such as in tracking and intercepting moving objects, the brain must overcome pervasive sensorimotor delays (Nijhawan, 2008; Franklin and Wolpert, 2011). Although it has been proposed that compensating for these inherent delays is based on bottom-up sensory extrapolation (e.g., the flash-lag effect, Nijhawan, 1994; Nijhawan and Wu, 2009), the prevalent view of sensorimotor control posits that action planning relies on forward models based on an intimate interplay between sensory inflow and motor outflow, rather than a hierarchical transformation from extrinsic stimuli to intrinsic muscular activity (Wolpert et al., 1995; Shadmehr and Mussa-Ivaldi, 2012).

## Forward Model for Predictive Sensorimotor Control

Movement planning predominately arises from an internal prediction of future states of body and environment, instead of merely relying on sensory responses. Since the emergence of the concept of forward models, important advances have been made in understanding how efference copies of motor commands are routed back to sensory structures for internally monitoring movement (Sommer and Wurtz, 2008). Those signals, widely referred as corollary discharges, have been observed across different species at many levels, including the cerebral cortex, spinal cord, cerebellum and muscle spindles (Crapse and Sommer, 2008). Through such wide-spread feedback in the form of closed sensorimotor loops, the brain is able to distinguish external motion from self-generated movements (Angelaki and Cullen, 2008), update sensory representations (Duhamel et al., 1992) and motor execution (Azim et al., 2014), and optimize active sensation (Kleinfeld and Deschênes, 2011). However, it is unclear where and how re-afferent signals are integrated with sensory inputs to form forward predictions leading to future movements, rather than solely monitoring them.

**Figure 1:**
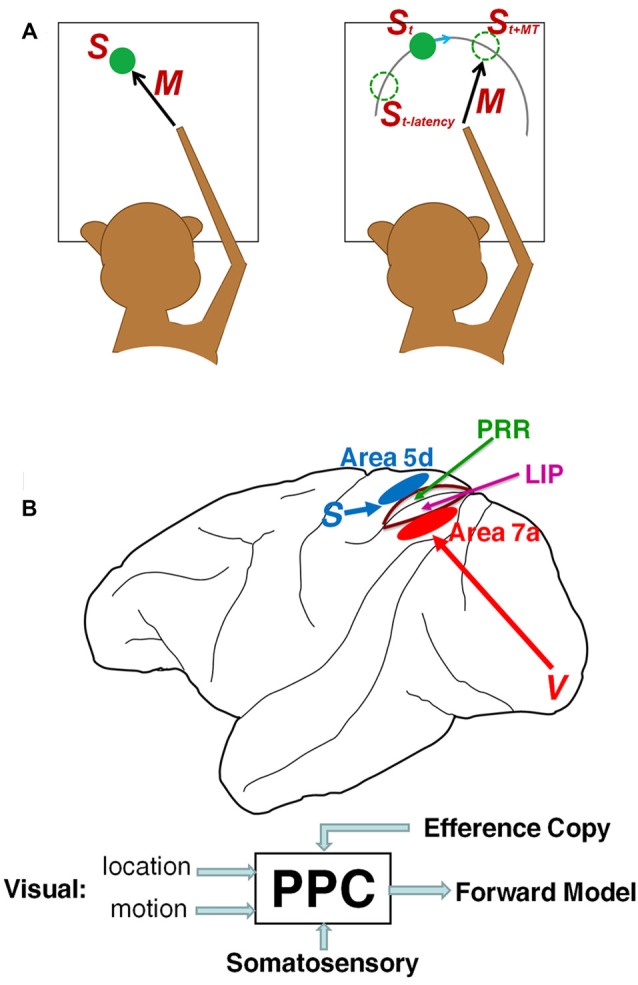
**Sensorimotor transformation and posterior parietal cortex (PPC). (A)** Unlike movement to a static target (left) in which motor parameters are tightly linked to a fixed stimulus location, in flexible interception (right) the brain not only compensates for sensory latency to estimate current stimulus location *S*_t_, but also predicts the future target location at reach offset *S*_t+MT_ to direct the intercepting movement. **(B)** The PPC might play a proactive role in predictive sensorimotor control by integrating visual (target location and motion), somatosensory and efference copy signal to form forward models of future object and body states.

Most studies in sensorimotor neurophysiology have utilized reactive movements to stationary goals pre-defined by sensory cues (Figure 1A left), but this approach is fundamentally incapable of determining whether the observed neural activity reflects sensory stimuli or predicts future states. Exploring the neural codes of a forward model demands the development of novel behavioral tasks that are highly dependent on predictive spatiotemporal transformations, such as interception (Figure 1A right), in which the movement is directed to a predicted future location of a moving target, as opposed to a static location explicitly specified by sensory cues. Interception has been widely investigated in numerous studies (see reviews Merchant and Georgopoulos, 2006; Zago et al., 2009). In the temporal domain, Merchant et al. (2004a,b) have shown that activity in both parietal and motor cortices encode estimations of arrival time (Tau-coupling, Lee, 1976) for precisely-timed interception at pre-determined destinations. In contrast to a wealth of data on temporal prediction, spatial extrapolation for interception has rarely been addressed, and consequentially little is known about its neural implementation.

During tracking and pursuing of moving stimuli, neuronal activity faithfully conveying the instantaneous or inferred target motion has been found in both cortical (e.g., Assad and Maunsell, 1995; Ferrera and Barborica, 2010) and subcortical areas (e.g., Cui and Malpeli, 2003; Ma et al., 2013). However, the neurons examined in these studies were primarily involved in the formation of up-to-date percepts, rather than in specifying the goals of the intercepting movements. For example, during catch-up saccades toward moving targets, pre-saccadic activity in the superior colliculus (SC) encodes retinal position error, instead of actual saccade metrics that take into account retinal slip (Keller et al., 1996). It is unclear how the time involved in actual generation and completion of the eye movements is taken into account.

## Plausible Role of the PPC in Forward Prediction

Internal prediction of the sensory consequences of extra-personal objects is, presumably, a high-order sensory representation that might be embodied in association areas. Moreover, forward prediction demands integrating different sources of sensorimotor information through a rich pattern of anatomical connectivity. As a crucial node incorporating visual, proprioceptive and efference copy information in a sensorimotor network (Andersen et al., 1997), the posterior parietal cortex (PPC) is a plausible candidate for mediating the fundamental relationship between sensory prediction and motor control (Figure 1B). Examining PPC activity during interceptive behaviors in dynamic environments might provide deep insights into how the brain constructs forward predictions for guiding movement, because the internal prediction of future target location can be inferred from the intercepting movements themselves. If the PPC underlies the forward model, PPC neurons should not merely convey the current states of body and object, but also should predict the future consequences of an impending movement. Indeed, accumulating evidence indicates that PPC activity predicts the sensory consequences of upcoming movements (Mulliken and Andersen, 2009). Thus, the PPC might fulfill the goal of forward prediction in sensorimotor transformations, and work in concert with inverse models in the motor cortex and the subcortical motor apparatus to implement them (Andersen and Cui, 2009).

Prediction is not only fundamental for motor control, but may also be crucial for many aspects of cognition, including sequential planning, decision making, social interaction, action understanding, imitation and mental practice. Therefore, forward prediction might offer a cohesive framework for understanding the neural basis of many related behaviors. For instance, neurological studies have shown that patients with lesion in the left PPC suffer from ideational apraxia (Buxbaum, 1998; Zadikoff and Lang, 2005). Although these patients appear normal for simple movements, they are impaired in associating objects with their purposes, and/or in generating complicated action sequences. A plausible interpretation is that the PPC damage interfered with the prediction of outcomes and consequences of forthcoming actions.

Growing evidence suggests that the PPC is composed of multiple functional subareas with distinct roles in the sensorimotor transformation (Andersen and Buneo, 2002; Cui, 2014). These include inferior parietal area 7a and superior parietal area 5d, as well as their upstream structures, and also the lateral intraparietal area (LIP) and parietal reach region (PRR; Figure 1B). Area 7a is the top structure in a dorsal visual hierarchy (Felleman and Van Essen, 1991; Bastos et al., 2014), with rich inputs from motion-sensitive extrastriate areas (Felleman and Van Essen, 1991). Its activity is modulated by world-centered gain fields (Snyder et al., 1998) and other top-down inputs from the prefrontal cortex (Crowe et al., 2013), hippocampus and cerebellum (Clower et al., 2001). Consequently, it is ideally positioned to represent the visual prediction of behaviorally relevant objects achieved through sensorimotor learning.

As a counterpart of area 7a, area 5 initially was thought of as a high-level somatosensory area (even sometimes referred to as S3) that conveys more abstract information about combined joint angles (Sakata et al., 1973). However, subsequent studies on behaving monkeys have shown that area 5 neurons convey kinematic information (Ashe and Georgopoulos, 1994; Kalaska and Crammond, 1995) for component arm movements (Li and Cui, 2013) in body-related coordinates (Johnson et al., 1996; Bremner and Andersen, 2012) by integrating visual and somatosensory inputs according to the behavioral context (Brunamonti et al., 2016). Given its close linkages to S1 and M1/PMd, area 5 might predict the somatosensory consequence of an upcoming arm movement during interception, with its neural activity primarily associated with intrinsic kinematic characteristics, such as limb trajectories, speed profiles and joint angles.

To investigate neural activity when movements are directed by anticipated sensory outcomes, rather than by current perceived stimulus locations, we have recorded parietal activity from monkeys performing a flexible manual interception task involving dynamic sensory-motor contingencies. In this paradigm, the monkey initiates a trial by positioning a hand at the center of a touch screen. A peripheral target moving at an angular velocity of 0 (control), 120, or 240°/s in a circular path appears in one of eight locations spaced at 45°. The targets, which could be moving either clockwise or counter clockwise, is visible for 1 s, have to be intercepted by a hand movement within this interval. During interception, a hand movement should be planned toward the anticipated target location at interception to accomplish the task. Therefore, if area 7a predicts the visual consequences of the upcoming interception, its pre-movement activity should encode movement destination, rather than instantaneous stimulus location. Preliminary data support this idea (Li et al., 2014). They suggest that movement-directional tuning curves of pre-movement activity are invariant, but stimulus-directional tuning curves significantly shift as functions of target speed. Partial correlation analysis demonstrates that PPC activity is more pronounced for the reaching direction than for the stimulus location, suggesting an intimate role in forward prediction and motor planning.

## Decoding PPC Activity for Feedforward Prosthetic Control

Over the past two decades, intracortically-based neuroprosthetics have emerged as promising approaches to restoring sensorimotor function for severely disabled patients suffering from nervous diseases or injuries. Based on neural activity recorded from chronically implanted multi-channel electrode arrays, intended movements have been successfully decoded as command signals and used to manipulate a robotic device or a cursor on a computer screen to replace the lost motor function of a paralyzed limb (Serruya et al., 2002; Carmena et al., 2003; Musallam et al., 2004; Hochberg et al., 2006; Santhanam et al., 2006; Velliste et al., 2008; Aflalo et al., 2015), even enabling it bimanually (Ifft et al., 2013) or bi-directionally (Ethier et al., 2012). Although such brain-machine interfaces (BMIs) have succeeded in continuously driving prosthetic arms, the achieved performances in movement speed, straightness and smoothness still fall short of widespread clinical applicability. Unlike natural movements planned in a feedforward manner, brain-controlled prosthetic devices demand continuous guidance of decoded neural signals, largely relying on visual feedback during movement execution. Furthermore, in a dynamic world, it is unlikely that closed-loop prosthetic systems depending on sensory feedback control are feasible for capturing moving objects.

**Figure 2:**
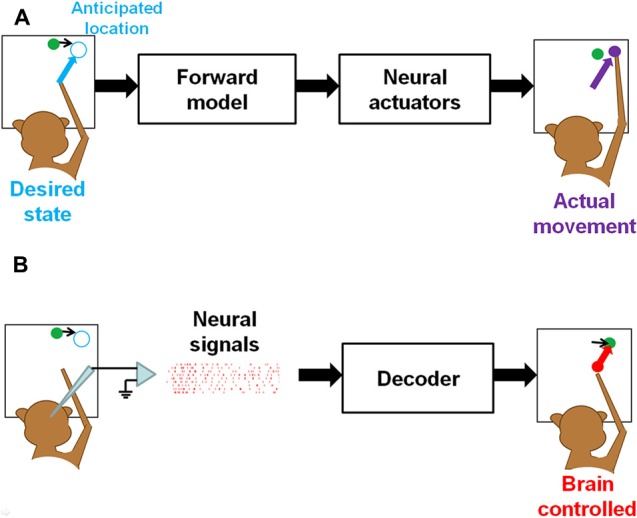
**Feedforward control of manual interception in naturalistic (A) and brain-controlled (B) conditions. (A)** In a manual interception task, the brain first predicts the future target location at movement offset, and then directs the arm (end-effector) toward it through open-loop control of musculoskeletal system. **(B)** In brain-controlled interception, predictive PPC activity is decoded to an intended motor goal, and used as a control signal to direct a cursor on a computer screen or a robotic actuator toward the future location at interception, as opposed to actually reaching for it with the hand.

Clinically viable BMI systems that enable real-time interactions with dynamic environments demand translation of predictive neural activity into desired motor goals to guide ballistic movements, as in natural interception (Figure 2A). Since our ongoing research has indicated that pre-movement activity in the PPC is informative of the intercepting movement, it is, in principle, possible to decoded this activity and utilize it as a predictor of an upcoming movement destination. Based on the decoded endpoint position, a computer cursor or artificial limb could be moved by a goal-directed open-loop controller (Figure 2B). After the desired endpoint is extracted from the PPC activity by an optimal estimation decoder (i.e., population vector, machine learning algorithm), the artificial actuator will be driven toward the intended movement goal while receiving no further brain controls until landing. If the actuator is a computer cursor, it will move to the goal in a bell-shaped speed profile, as described by Fitts’ law. If the actuator is a high-degree of freedom (DOF) robotic limb, it will be driven by a goal-directed pre-coded program fitted to its own mechanics, rather than by biomimetic kinematics. Of course, how the central nervous system compensates for prosthetic motor errors through learning and adaptation to archieve proficient BMI control is a challenging problem.

## Discussion

Even if predictive activity embodied in the PPC can provide useful neural signals for open-loop prosthetic control of movements, important issues remained to be addressed. Predictive sensorimotor control engages a highly distributed cortical network, so the PPC alone probably is inadequate for directing comprehensive and adaptable motor repertoires. Preferably, a PPC-based controller should be integrated with neural signals from motor areas to implement timely and accurate control of rapidly-moving actuators. Furthermore, because successful interception requires not only accurate spatial prediction, but also precise timing (Merchant and Georgopoulos, 2006; Zago et al., 2009), dynamic BMIs based on the PPC should also incorporate temporal information conveyed in the motor and premotor cortex (Merchant and Georgopoulos, 2006; Merchant et al., 2009, 2011).

Despite the importance of forward models, movement can still be modified by sensory feedback during execution (Todorov and Jordan, 2002; Scott, 2004). According to the behavioral context, natural interceptions might adopt reactive, predictive, or mixed strategies (Port et al., 1997; Merchant et al., 2003). Thus, prosthetic movements directed to an endpoint decoded from PPC signals could be corrected online by an artificial proprioceptive feedback via intracortical microstimulation (Dadarlat et al., 2015), probably in the deceleration phase.

In summary, BMIs capable of implementing predictive sensorimotor control in dynamic circumstances require systematic integration of modern motor neuroscience and numerous sophisticated techniques. If successful, such an approach may allow paralyzed patients to bypass their immobilized bodies, and interact with the dynamic external world.

## Author Contributions

HC conceived the study and wrote the article.

## Conflict of Interest Statement

The author declares that the research was conducted in the absence of any commercial or financial relationships that could be construed as a potential conflict of interest.
